# Response assessment of post-treatment head and neck cancers to determine further management using NI-RADS (Neck Imaging Reporting and Data System): a subgroup analysis of a randomized controlled trial

**DOI:** 10.3389/fonc.2023.1200366

**Published:** 2023-09-21

**Authors:** Abhishek Mahajan, Himangi Unde, Nilesh P. Sable, Shreya Shukla, Richa Vaish, Vijay Patil, Ujjwal Agarwal, Archi Agrawal, Vanita Noronha, Amit Joshi, Akhil Kapoor, Nandini Menon, Jai Prakash Agarwal, Sarbani Ghosh Laskar, Anil Keith Dcruz, Pankaj Chaturvedi, Prathamesh Pai, Swapnil Ulhas Rane, Munita Bal, Asawari Patil, Kumar Prabhash

**Affiliations:** ^1^ Department of Radiodiagnosis, The Clatterbridge Cancer Centre, University of Liverpool, Liverpool, United Kingdom; ^2^ Department of Radiodiagnosis and Imaging, Tata Memorial Hospital, Homi Bhabha National Institute, Mumbai, India; ^3^ Department of Head and Neck Surgery, Tata Memorial Hospital, Homi Bhabha National Institute, Mumbai, India; ^4^ Department of Medical Oncology, Tata Memorial Hospital, Homi Bhabha National Institute, Mumbai, India; ^5^ Department of Nuclear Medicine, Tata Memorial Hospital, Homi Bhabha National Institute, Mumbai, India; ^6^ Department of Radiation Oncology, Tata Memorial Hospital, Homi Bhabha National Institute, Mumbai, India; ^7^ Department of Pathology, Tata Memorial Hospital, Homi Bhabha National Institute, Mumbai, India

**Keywords:** head neck cancer imaging, head neck cancer follow-up, computed tomography, PET, MRI, NIRADS, surveillance, post treatment response

## Abstract

**Objective:**

Interpreting complex post-treatment changes in head and neck cancer (HNC) is challenging with further added perplexity due to variable interobserver interpretation and hence evolved the NI-RADS lexicon. We evaluated the accuracy of NI-RADS in predicting disease status on 1st post-treatment follow-up CECT in a homogenous cohort of those who received only chemoradiation.

**Methods:**

Retrospective analysis of imaging was done for LASHNC patients who received radical chemoradiation in an open-label, investigator-initiated, phase 3 randomized trial (2012-2018) randomly assigned to either radical radiotherapy with concurrent weekly cisplatin (CRT) or CRT with the same schedule plus weekly nimotuzumab (NCRT). 536 patients were accrued, and 74 patients who did not undergo PET/CECT after 8 weeks post-CRT were excluded. After assessing 462 patients for eligibility to allocate NI-RADS at primary and node sites, 435 cases fell in the Primary disease cohort and 412 cases in the Node disease cohort. We evaluated sensitivity, disease prevalence, the positive and negative predictive value of the NI-RADS lexicon, and accuracy, which were expressed as percentages. We also prepared flow charts to determine concordance with allocated NI-RADS category and established accuracy with which it can identify disease status.

**Results:**

Out of 435 primary disease cohort, 92%, 55%, 48%,70% were concordant and had 100%, 72%, 70%, 82% accuracy in NI-RADS1 (n=12), NI-RADS2 (n=261), NIRADS3 (n=105), and NI-RADS 4 (n=60) respectively. Out of 412 nodes disease cohort, 95%, 90%, 48%, 70%were concordant and had 92%, 97%, 90%, 67% accuracy in NI-RADS1 (n=57), NI-RADS2 (n=255), NI-RADS3 (n=105) and NI-RADS4 (n=60) respectively. % concordance of PET/CT and CECT across all primary and node disease cohorts revealed that PET/CT was 91% concordant in primary NI-RADS2 as compared to 55% concordance of CECT whereas concordance of CECT was better with 57% in primary NI-RADS3 cohort as compared to PET/CT concordance of 41%.

**Conclusion:**

The accuracy with which the NI-RADS lexicon performed in our study at node sites was better than that at the primary site. There is a great scope of research to understand if CECT performs better over clinical disease status in NI-RADS 3 and 4 categories. Further research should be carried out to understand if PET/CECT can be used for close interval follow-up in stage III/IV NI-RADS 2 cases.

## Introduction

Imaging has become an integral part of the multidisciplinary management of head and neck cancer (HNC) patients to optimize prognosis and preserve function (1). Due to the availability of a wide range of multimodality treatment options resulting in complex post-treatment changes, interpreting them on imaging becomes challenging (2–4). Variable interobserver interpretation of imaging further adds perplexity in guiding the treating physicians (5). The ACR formed the Neck Imaging Reporting and Data Systems (NI-RADS) Committee in August 2016 to standardize the reporting of surveillance imaging to guide the management of patients with treated HNC. The NI-RADS was originally developed for surveillance contrast-enhanced CT (CECT) imaging with or without positron emission tomography (PET) in patients with treated HNC. It was aimed to simplify the communication between radiologists and referring clinicians and provide management guidance for specific levels of suspicion (5, 6). The ACR-NIRADS lexicon has been constantly evolving over the last few years, with the last revised version of the PET/CT lexicon published in January 2021, followed by the MRI lexicon in November 2021. The primary tumor site and neck lymph nodes are scored separately based on imaging suspicion of recurrence as NI-RADS1–4. NI-RADS1 indicates no evidence of recurrence, whereas NI-RADS4 is already known/biopsy-proven recurrence. New or enlarging discrete soft tissue with intense differential enhancement with or without bone erosion (7) qualifies for NI-RADS3 category for primary site. Enlarging node(s) with new necrosis or gross extranodal extension (ENE) (8) qualifies for NI-RADS3 category for neck nodes, both of which indicate biopsy of the area of concern. The first post-treatment imaging examination is important to predict prognosis as surgery has 70% 2-year relapse-free survival rate in early-stage (stage I/II) recurrence as compared to those with resectable advanced-stage HNSCC (stage III/IV), which have 25% 2-year relapse-free survival rate (9). Familiarity with the imaging characteristics of post-treatment changes and of the potential complications caused by surgery and irradiation and an ability to differentiate these findings from tumor recurrence are essential for post-treatment surveillance and follow-up management of patients with head and neck cancer (10). The post-radiation therapy imaging features of early reactions on CT and MR are thickening of the skin to radiation therapy and platysma, reticulation of the subcutaneous fat, edema and fluid in the retropharyngeal space, increased enhancement of the major salivary glands, thickening and increased enhancement of the pharyngeal walls, and thickening of the laryngeal structures (11, 12). As there is an extensive sundry of imaging features for various post-treatment changes in early-stage and late-stage HNC patients (1) including post-resection with or without flap reconstruction leading to altered anatomy, postoperative complications, post-chemo-radiation, and recurrence (9), it necessitated evaluating the accuracy of NI-RADS under a specific treatment condition. Due to the varied imaging features of these post-treatment changes, NI-RADS serves as a practical guide to interpreting radiologists which simplifies categorizing of the imaging features linked to specific levels of suspicion (5, 6). Knowledge of preferred imaging modalities in various settings and a standard protocol for optimal image acquisition are necessary (6, 13). While early detection of recurrence might yield improved survival outcomes, imaging before 8–12 weeks might be disadvantageous due to the presence of post-treatment inflammation (14, 15). Interpretation of post-treatment imaging may pose a challenge to inexperienced radiologists. Such scans should be reviewed by experienced in-house sub-speciality radiologists if the scan and reporting were done in another diagnostic center/hospital (16). Our hospital is a tertiary cancer care institute which caters to a very large number of HNC patients annually. Hence, there is a great need for standardized radiology reporting with a linked management algorithm that will help to reduce inter-reader variability and guide further management in such post-treated HNC patients. In this study, we have evaluated the accuracy of NI-RADS in predicting disease status on first post-treatment follow-up CECT in a homogenous cohort of those who received only chemoradiation.

## Materials and methods

### Materials

**Figure 1:**
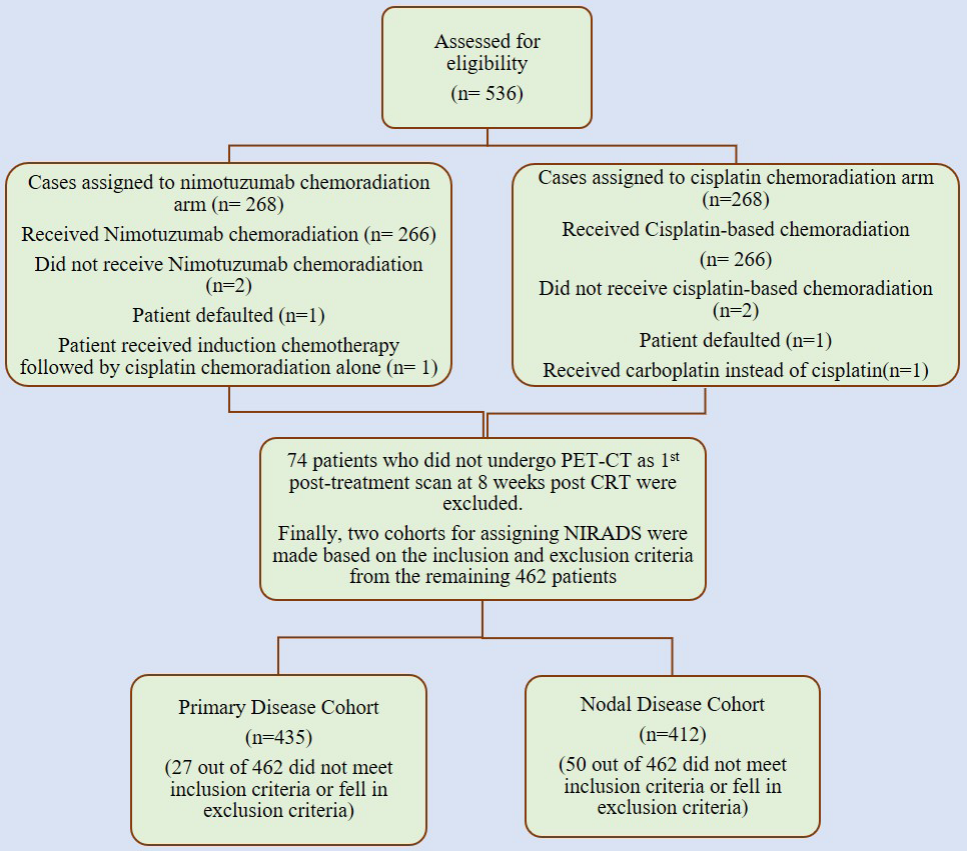
Consolidated algorithm of the nimotuzumab plus cisplatin-radiation versus cisplatin-radiation arm showing the case selection for allocation of NI-RADS.

This was a retrospective analysis of imaging done for patients who were enrolled in a prospective randomized control trial study at our institute with non-metastatic, locally advanced stage III or IV HNSCC and who were fit for radical chemoradiation. These patients were randomized 1:1 to receive either radical radiotherapy (66–70 grays) with concurrent weekly cisplatin (30 mg/m^2^) or the same schedule of chemoradiotherapy (CRT) with weekly nimotuzumab (200 mg) (NCRT) at our tertiary care cancer institute between 1 January 2012 and 31 July 2017. The inclusion criteria included age of more than 18 years, treatment-naive head neck squamous cell carcinoma patients (oral, oro-pharynx, hypopharynx, and larynx) who underwent post-treatment positron emission tomography/contrast-enhanced computed tomography (PET/CECT) performed 8 weeks after radical CRT whose scans are available on Picture Archiving and Communication System (PACS) in our institute and with a minimum follow-up period of 12 months or had proven clinical/radiological recurrence (primary or nodal) prior to it. The key exclusion criteria included primary tumors with non-squamous cell histopathological report (HPR), patients with tumors originating in the nasopharynx, salivary gland, or nasal cavity, those who had received immunotherapy or prior radiotherapy to the head neck region, patients with imaging reviewed at our institute without available DICOM data, and patients who initially presented with distant metastasis (17). Other important exclusion criteria included concurrent second primary cancers, patients without adequate treatment details/clinical follow-up data and lastly suboptimal CECT component of PET CT to assign NIRADS either due to motion artefacts or metallic streak artefacts of the dentures. Hence, for our retrospective study, we screened patients from these 536 cases, out of which 74 patients who did not undergo PET/CECT at 8 weeks post-CRT were excluded. We assessed these 462 patients for eligibility in our study to allocate NI-RADS at primary and node sites. **Figure 1** shows the consolidated algorithm of the study.

### Study methodology

The prospective randomized control trial was approved by the institutional ethics committee and was registered with the clinical trial registry of India (CTRI/2014/09/004980). This was an investigator-initiated, randomized controlled trial. All patients underwent standard study protocol for evaluation and treatment. The detailed inclusion and exclusion criteria of the trial are mentioned in the supplement of the main published article of the trial (17). Head and neck examination, dental examination, blood tests, chest radiograph, and electrocardiogram before randomization of selected patients were performed. Randomization for five factors were carried out by an independent statistician, and patients have randomized 1:1 to either the cisplatin radiation arm (CRT) or the nimotuzumab–cisplatin radiation arm (NCRT) (17). The risk factors included the site of malignancy (oropharynx vs. larynx-hypopharynx), overall stage of disease (stage III vs. IV), age (≤60 vs. >60 years), and T stage. The data of the patients were obtained from electronic medical records, including age, sex, clinical nodal positive status, tumor site and clinical TNM staging (7th edition of AJCC cancer staging), which were further used as clinical variables for analysis. Both arms administered high-dose, curative radiotherapy for 6.5 to 7 weeks (17). Using a standard two-dimensional (2D) technique, a three-dimensional (3D) conformal technique, or intensity-modulated radiotherapy with megavoltage radiation, irradiation was planned (17). Local tumor and lymph node disease were treated with 70 grays (Gy), in 2 Gy per fraction, at 5 days per week. A dose of up to 46 to 50 Gy was planned for the uninvolved nodal regions of the neck. Other altered fractionation schedules were permitted if the biological equivalent dose for tumor control was similar to 70 Gy at 2 Gy per fraction. Quality check was done, and plans and doses were cross-verified and confirmed by the radiation oncology teams (17). Cisplatin was dosed at 30 mg/m^2^ weekly during radiation along with supportive medication in both arms (17). Weekly nimotuzumab was given in the NCRT arm intravenously as a 200-mg dose in 250 mL of normal saline over 60 min without any premedication (17). At 8 weeks post-CRT, these patients underwent PET/CECT for response assessment.

So, we evaluated the imaging of these patients in our retrospective study after approval by the institutional ethics committee. We screened patients from these 536 cases, out of which 74 patients who did not undergo it at 8 weeks post-CRT were excluded. We assessed these 462 patients for eligibility in our study to allocate NI-RADS at primary and node sites, and after applying inclusion and exclusion criteria, we had a primary disease cohort with 435 cases and a node disease cohort with 412 cases.

Data from the patients were obtained from electronic medical records. The patient’s age, sex, clinical TNM staging, tumor location, histopathological grade of primary and nodal sites if available, and post-treatment disease status on clinical and/or imaging follow-up were noted from electronic medical records which were further used as a clinical variable for analysis. On follow-up, the HPR of patients who underwent biopsy/fine needle aspiration cytology/surgery was noted wherever available. As all the patients who were included in the study had undergone PET/CECT as first post-treatment scan, PET findings at the primary and nodal sites were also noted. Similarly, on follow-up imaging whichever patients underwent PET/CECT, PET and CECT findings at the primary and nodal sites were noted. The disease management group radiologist with more than 13 years of experience who was responsible for assigning the NI-RADS category for primary and nodal sites was blinded to the pre-treatment and post-treatment clinical details and the HPR report of the patients. He reviewed only the non-fused whole-body contrast-enhanced computed tomography (WB-CECT) series of the PET/CECT scan done at 8 weeks post-treatment and assigned the NI-RADS category to primary and nodal sites, respectively. The NI-RADS categories were assigned to the non-fused WB-CECT series of the PET/CECT as per the NI-RADS version of 2018 published by the American College of Radiology.

**Figure 2:**
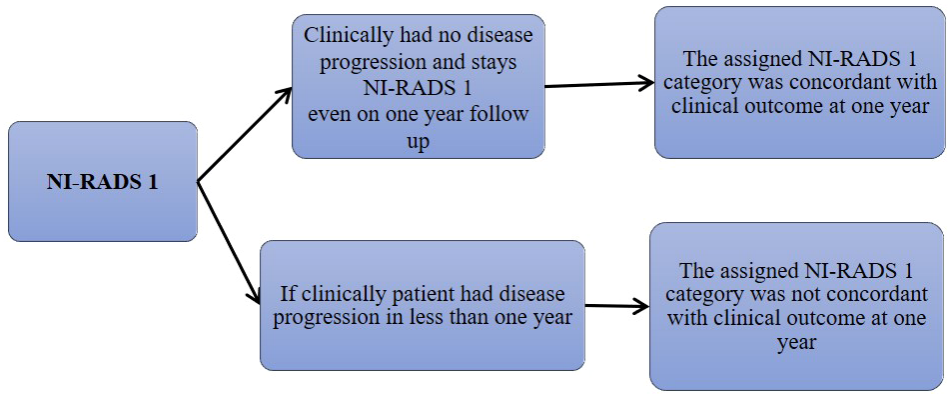
Flow chart describing process in which allocation of NI-RADS1 to Primary and Nodal disease cohort was considered concordant.

**Figure 3:**
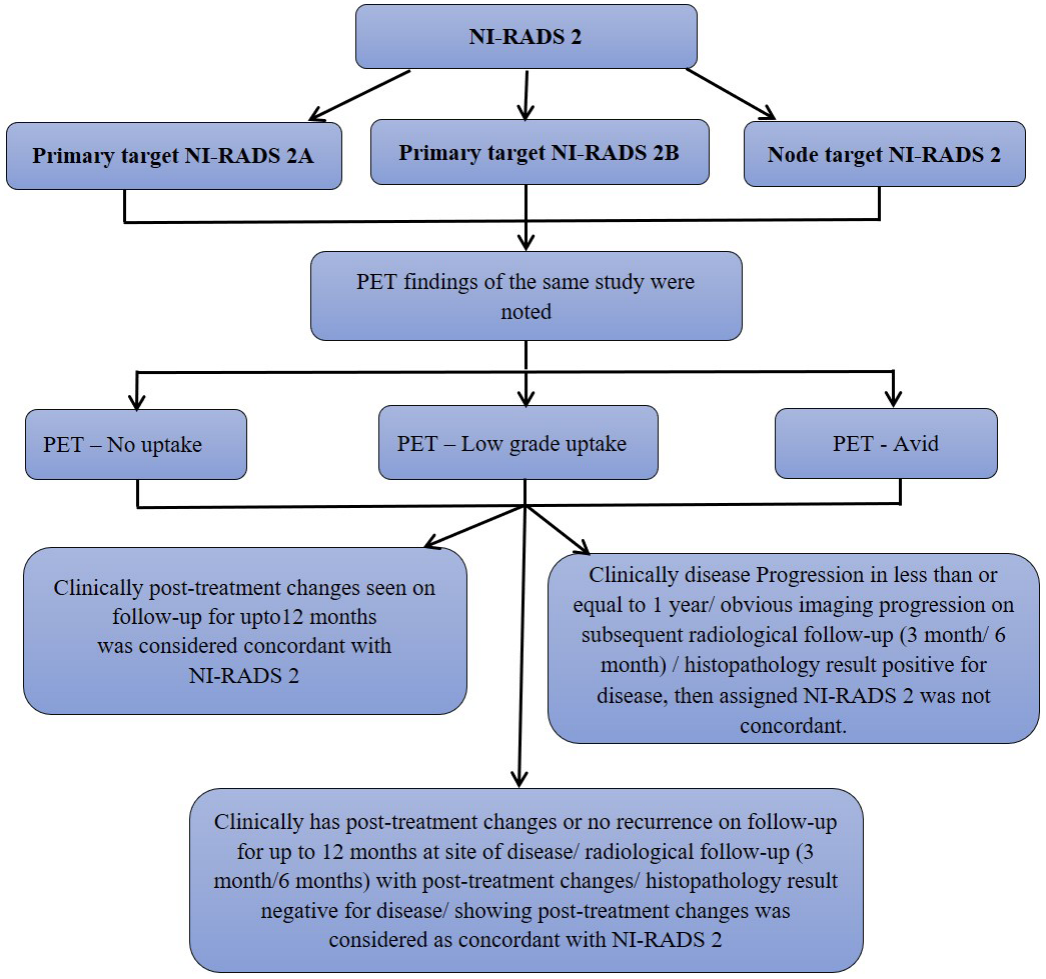
Flow chart describing process in which allocation of NI-RADS2 to Primary and Nodal disease cohort was considered concordant.

**Figure 4:**
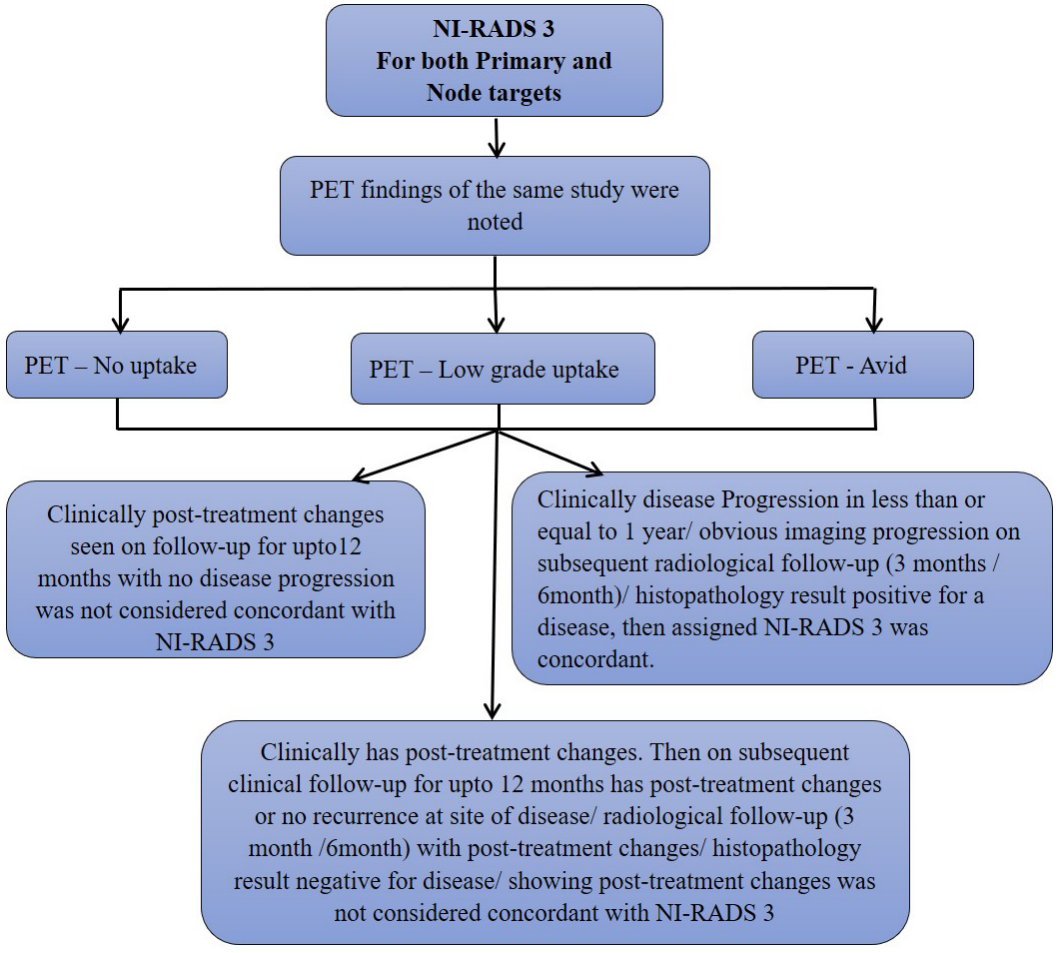
Flow chart describing process in which allocation of NI-RADS3 to Primary and Nodal disease cohort was considered concordant.

**Figure 5:**
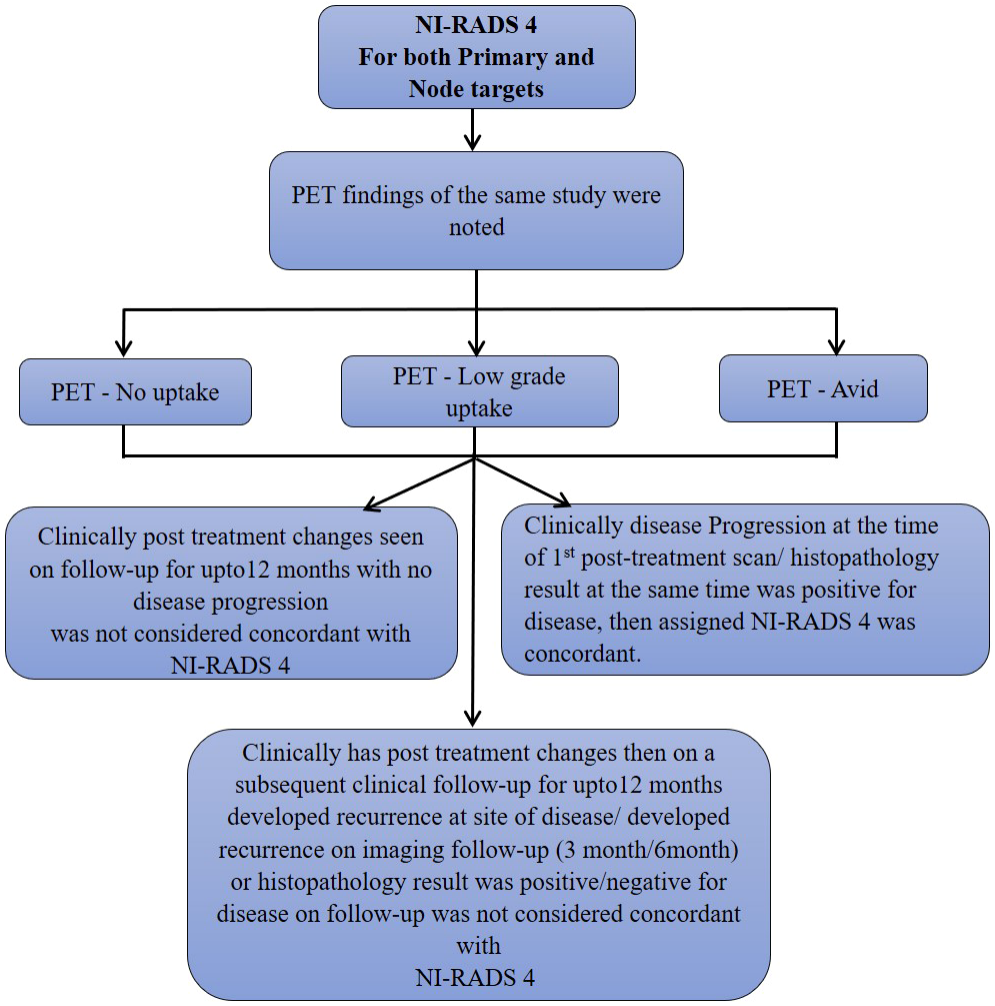
Flow chart describing process in which allocation of NI-RADS4 to Primary and Nodal disease cohort was considered concordant.

We also prepared flow charts (**Figures 2**–**5**) to determine concordance with the allocated NI-RADS category and established accuracy with which it can identify disease status. The post-treatment clinical outcome and available imaging and histopathologic results for each patient who was assigned the NI-RADS category were noted from electronic medical records for a period of 1 year.

### Statistical analysis

Diagnostic accuracy parameters like sensitivity, specificity, disease prevalence, positive and negative predictive value as well as accuracy expressed as percentages were calculated as a test to post-treatment disease status on imaging *versus* clinical outcome as gold standard and/or HPR as gold standard wherever available. Statistical analysis was performed using SPSS version 25 (Statistical Package for the Social Sciences).

## Results

### Patient characteristics

Out of the primary disease cohort with 435 cases, 12 cases were labeled NI-RADS1, five patients were labeled NI-RADS2A, and 256 patients were labeled NI-RADS2B. To simplify the statistical analysis, both NI-RADS2A and 2B were clubbed together, forming a total of 261 cases as NI-RADS2, 105 cases labeled as NI-RADS3, and 57 cases labeled as NI-RADS4. Likewise, out of the node disease cohort with 412 cases, 57 cases were labeled NI-RADS1, 255 cases were labeled NI-RADS2, 40 cases were labeled NI-RADS3, and 60 cases were labeled NI-RADS4. Patient demographics, clinical treatment, imaging, and histopathological details are mentioned in **Tables 1**, **2**.

**Table 1:** Demographics, Clinico-Radio-Pathological Details of Primary Site Cohort.

Primary disease cohort	NI-RADS1	NI-RADS2	NI-RADS3	NI-RADS4	Total
Male	10	219	96	52	**377**
Female	2	42	9	5	**58**
**Total**	**12**	**261**	**105**	**57**	**435**
Cisplatin Arm	6	133	51	24	**214**
Cisplatin + Nimotuzumab Arm	6	128	54	33	**221**
Primary Tumour Site					
Hypopharynx	4	53	20	10	**87**
Larynx	2	89	33	8	**132**
Oropharynx	**5**	**119**	**52**	**39**	**215**
Oral cavity	1	0	0	0	**1**
HPR Grade					
Not Documented	0	157	67	35	**259**
Well differentiated	4	3	1	1	**9**
Moderately Differentiated	3	32	15	6	**56**
Poorly Differentiated	**5**	**69**	**22**	**15**	**111**
T Stage					
T1	1	12	1	1	**15**
T2	3	42	11	9	**65**
T3	**5**	**134**	45	16	**200**
T4a	2	64	**46**	**30**	**142**
T4b	1	9	2	1	**13**
Stage of Disease					
III	2	97	29	9	**137**
IVA	**8**	**150**	**75**	**47**	**280**
IVB	2	14	1	1	**18**
PET Imaging					
No uptake	**11**	**216**	**62**	10	**299**
Low Grade Uptake	0	21	18	4	**43**
Avid	1	24	25	**43**	**93**
Clinical Progression (less than or equal to 1year)					
Yes	1	108	51	41	**201**
No	11	153	54	16	**234**
Imaging Progression (less than or equal to 1year)					
Yes	1	34	19	12	**66**
No	10	200	60	16	**286**
Progression on Histopath (less than or equal to 1year)					
Yes	**1**	**39**	**26**	**23**	**89**
No	1	15	14	7	**37**
Not done	10	207	65	27	**309**

**Table 2:** Demographics, Clinico-Radio-Pathological Details of Node Site Cohort.

Nodal disease cohort	NI-RADS1	NI-RADS2	NI-RADS3	NI-RADS4	Total
Male	48	221	35	52	**356**
Female	9	34	5	8	**56**
**Total**	**57**	**255**	**40**	**60**	**412**
Cisplatin Arm	27	127	16	33	**203**
Cisplatin + Nimotuzumab arm	30	128	24	27	**209**
Node Tumour Site					
Hypopharynx	13	49	4	11	**77**
Larynx	20	92	6	11	**129**
Oropharynx	**24**	**114**	**29**	**38**	**205**
Oral cavity	0	0	1	0	**1**
HPR Grade					
Not Documented	41	152	20	36	**249**
Well differentiated	0	3	1	1	**5**
Moderately Differentiated	**9**	35	5	6	**55**
Poorly Differentiated	7	**65**	**14**	**17**	**103**
N Stage					
N0	**23**	**89**	1	1	**114**
N1	13	57	5	9	**84**
N2a	1	2	5	7	**15**
N2b	12	52	**19**	19	**102**
N2c	7	53	8	**22**	**90**
N3	1	2	2	2	**7**
Stage of Disease					
Stage III	20	103	3	7	**133**
Stage IVA	**32**	**145**	**35**	**50**	**262**
Stage IVB	5	7	2	3	**17**
PET Imaging					
No uptake	**49**	**213**	**18**	14	**294**
Low Grade Uptake	6	28	7	12	**53**
Avid	2	14	15	**34**	**65**
Clinical Progression (less than or equal to 1 year)					
Yes	3	26	25	26	**80**
No	54	229	15	34	**332**
Imaging Progression (less than or equal to 1 year)					
Yes	4	29	12	29	**74**
No	49	205	20	19	**293**
Progression on Histopath (less than or equal to 1 year)					
Yes	5	29	16	29	**79**
No	5	18	5	7	**35**
Not done	47	208	19	24	**298**

**Figure 6:**
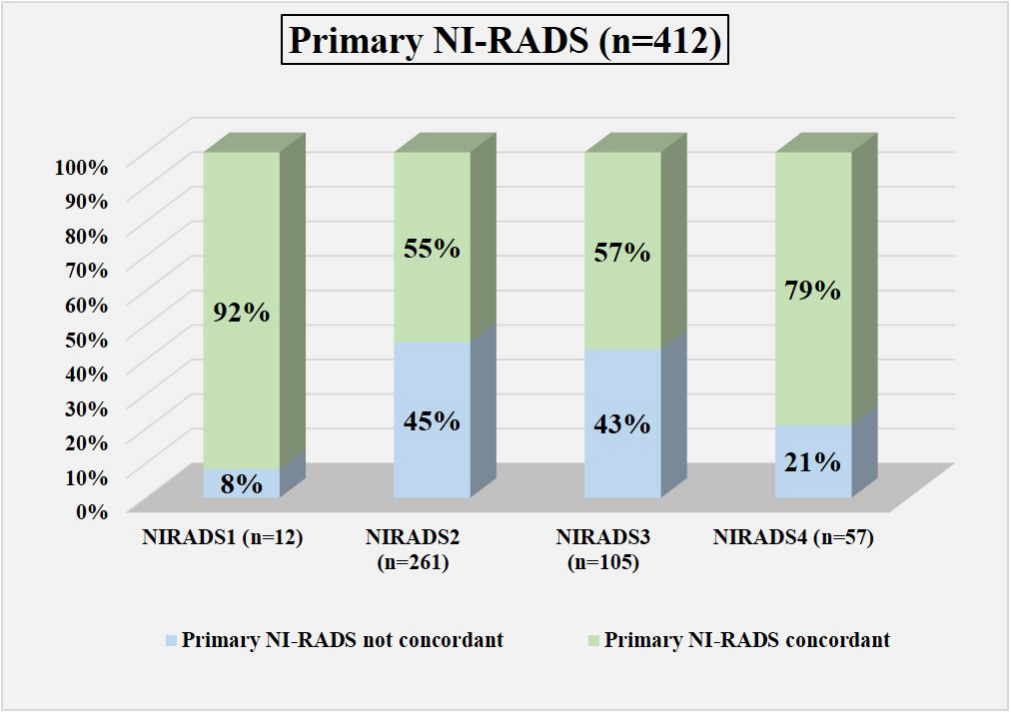
Bar diagram showing the percentage of cases in which the assigned Primary NI-RADS category was concordant.

**Figure 7:**
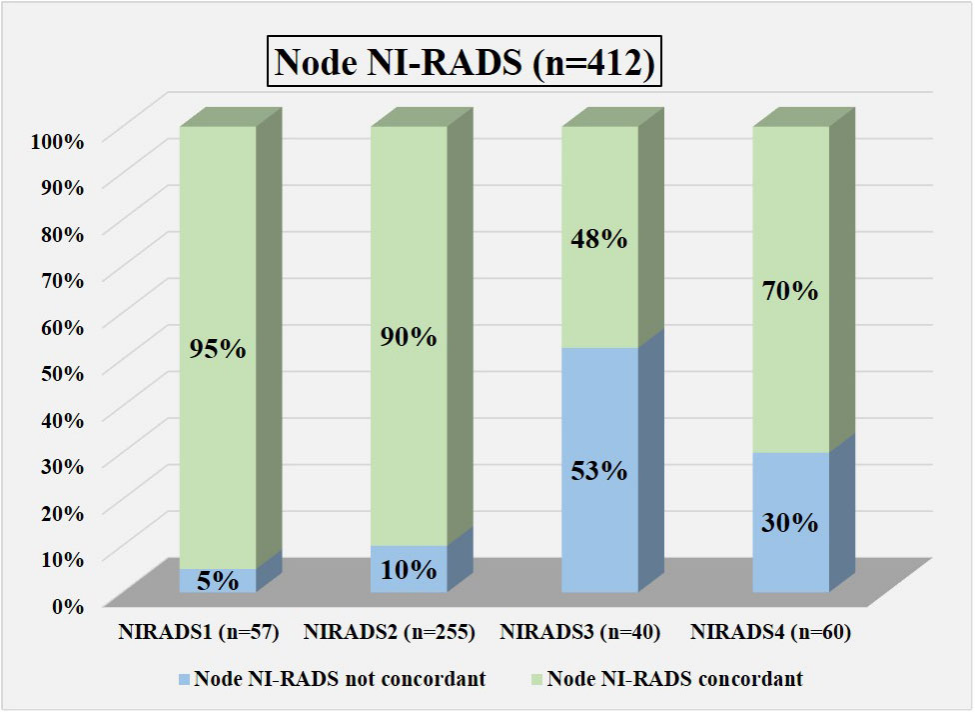
Bar diagram showing the percentage of cases in which assigned node NI-RADS category was concordant.

Based on the prepared flow charts (**Figures 2**–**5**), we further evaluated concordance with the allocated NI-RADS category for both the primary disease cohort (**Figure 6**) and the node disease cohort (**Figure 7**) and assessed the accuracy with which it can identify disease status.

### Primary disease cohort

In total, 12 were labeled NI-RADS1, out of which 92% were concordant and had 100% accuracy to identify no disease; 261 were labeled NI-RADS2, out of which 55% were concordant and had 72% accuracy to identify no disease; 105 were labeled NI-RADS3, out of which 57% were concordant and had 70% accuracy to identify the presence of disease; and 57 were labeled NI-RADS4, out of which 79% were concordant and had 82% accuracy to identify the presence of disease. We also assessed the positive predictive value and the negative predictive value for each assessed category as mentioned in **Figure 6**.

We also compared the %concordance of assessed NI-RADS on CECT with PET/CT findings and clinical assessment of disease status up to 1-year follow-up (**Table 3**). We found that the %concordance for assessed CECT NI-RADS1, PET/CT, and clinical assessment of disease status up to 1-year follow-up was equal (92%), and the %concordance for assessed CECT NI-RADS2, PET/CT (91%) was better than CECT (55%) and clinical assessment of disease status up to 1-year follow up (59%). The %concordance for assessed CECT NI-RADS3, CECT (57%) was better than the clinical assessment of disease status up to 1-year follow-up (49%) and PET/CT (41%). The %concordance for assessed CECT NI-RADS4 and concordance of CECT (79%) was nearly the same as PET/CT (75%) and slightly better than the clinical assessment of disease status up to 1-year follow-up (72%).

**Table 3:** Percent concordance of CECT, PET CT and Clinical disease status for respective Primary and Nodes NI-RADS.

COHORTS		No. of ca-ses	No uptake	low grade upta-ke	avid	% concorda-nce PET	% concorda-nce CECT	% Clinical concorda-nce	% Accuracy of NIRADS for CECT
Primary									
NI-RADS1 (12)	concordant	11	10	0	1	92%	92%	92%	100%
	not concordant	1	1	0	0	8%	8%	8%	
NI-RADS2 (261)	concordant	143	130	11	2	91%	55%	59%	72%
	not concordant	118	86	10	22	9%	45%	41%	
NI-RADS3 (105)	concordant	60	27	14	19	41%	57%	49%	70%
	not concordant	45	35	4	6	59%	43%	51%	
NI-RADS4 (57)	concordant	45	5	2	38	75%	79%	72%	82%
	not concordant	12	5	2	5	25%	21%	28%	
Nodes									
NI-RADS1 (57)	concordant	54	47	5	2	86%	95%	95%	92%
	not concordant	3	2	1	0	14%	5%	5%	
NI-RADS2 (255)	concordant	229	195	24	10	95%	90%	90%	97%
	not concordant	26	18	4	4	5%	10%	10%	
NI-RADS3 (40)	concordant	19	8	1	10	53%	48%	62.5%	90%
	not concordant	21	11	6	4	47%	53%	37.5%	
NI-RADS4 (60)	concordant	42	6	9	27	57%	70%	43%	67%
	not concordant	18	8	3	7	43%	30%	57%	

### Nodal disease cohort

In total, 57 were labeled NI-RADS1, out of which 95% were concordant and had 92% accuracy to identify no disease; 255 were labeled NI-RADS2, out of which 90% were concordant and had 97% accuracy to identify no disease; 40 were labeled NI-RADS3, out of which 48% were concordant; and had 90% accuracy to identify the presence of disease. A total of 60 were labeled NI-RADS4, out of which 70% were concordant and had 67% accuracy to identify the presence of disease. We also assessed the positive predictive value and the negative predictive values for each assessed category as mentioned in **Figure 7**.

We also compared the %concordance of assessed NI-RADS on CECT with PET/CT findings and clinical assessment of disease status up to 1-year follow-up (**Table 3**). We found that the %concordance for assessed CECT NI-RADS1, CECT and clinical assessment of disease status up to 1-year follow-up was equal (95%), while PET/CT was slightly lower (86%). The %concordance for assessed CECT NI-RADS2, PET/CT (95%) was slightly better than CECT (90%) and the clinical assessment of disease status up to 1-year follow-up (90%). The %concordance for assessed CECT NI-RADS3, clinical assessment of disease status up to 1-year follow-up (62.5%) was better than CECT (48%) and PET/CT (35%). The %concordance for assessed CECT NI-RADS4, CECT (70%) was better than PET/CT (57%) and clinical assessment of disease status up to 1-year follow-up (43%).

A line diagram showing the performance of NI-RADS in our primary disease cohort and nodes disease cohort are illustrated in **Figures 8**, **9**. The cases in which there was discordance/ concordance between the assigned NIRADS category and their final outcome have been discussed in **Supplementary Figures 1–8**.

**Figure 8:**
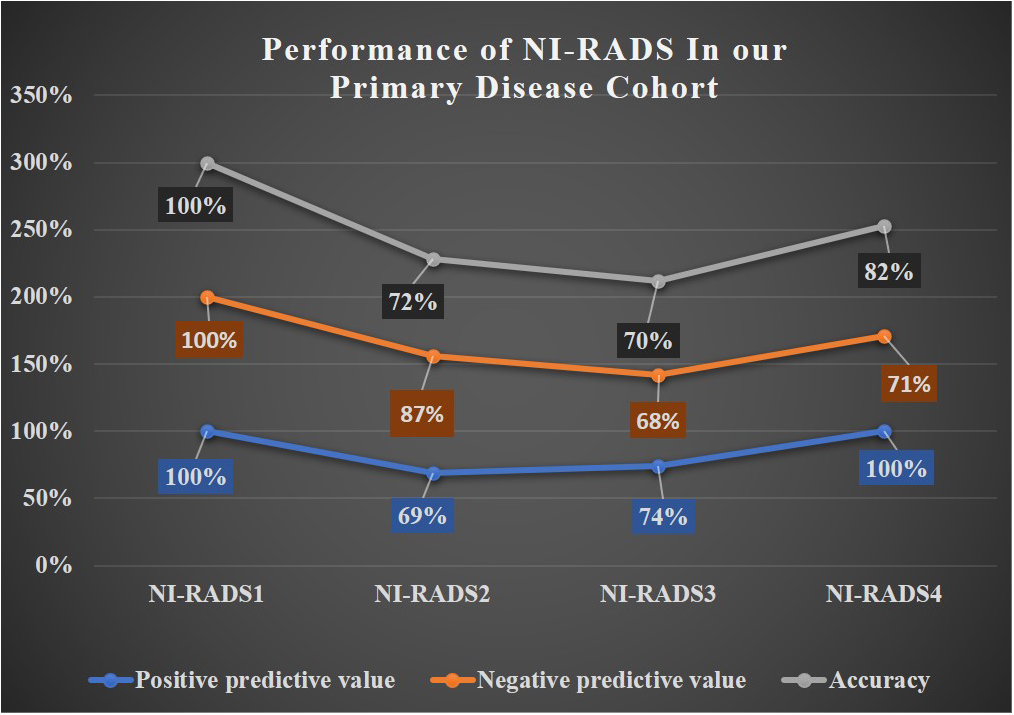
Line diagram showing performance of NI-RADS in our Primary Disease Cohort.

**Figure 9:**
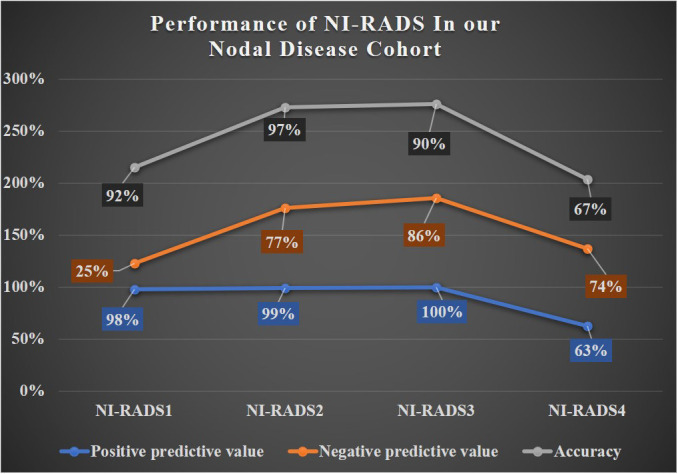
Line diagram Chart showing performance of NI-RADS in our Nodal Disease Cohort.

## Discussion

Treatment intensification with concurrent chemoradiation approaches, neoadjuvant chemotherapy, and altered fractionation regimens have improved treatment outcomes in patients with HNCs (18–25), though with increased early toxic effects (26, 27). As there is an exhaustive list of imaging features for various post-treatment changes in early-stage and late-stage HNC patients (1), including post-resection with or without flap reconstruction leading to altered anatomy, postoperative complications, post-chemo-radiation changes, and recurrence (28), it necessitated evaluating the accuracy of NI-RADS under a specific treatment modality. Hence, we evaluated the accuracy of NI-RADS in predicting disease status on the first post-treatment follow-up CECT in a homogenous cohort of those who received only chemoradiation (17).

The baseline performance of NI-RADS in our study suggested that node NI-RADS performed better across all categories, except NI-RADS4 with PPV of 63% and accuracy of 67%, while the performance of NI-RADS2 and NI-RADS3 category cases of the primary cohort was relatively similar in terms of PPV and accuracy, mostly due to complex imaging features at the post-treatment site, making it difficult to allocate appropriate NI-RADS. Here PET/CECT imaging and close interval follow-up can be used as a solving tool in NI-RADS2 category, and biopsy can be recommended for NI-RADS3 cases. Patients who are categorized as NI-RADS1 in the primary disease cohort can undergo their next surveillance imaging after 6 months with 100% PPV and accuracy to identify no disease and 100% NPV to identify the presence of disease. The positive predictive value for NI-RADS3 primary site lesions was lower (74%) than for the node site (100%); this is most likely due to the more complex imaging appearances at the primary site due to post-treatment changes.

The NI-RADS1 group of the node disease cohort, among whom two patients showed avidity and had no disease progression on follow-up, suggested that first post-treatment PET/CT may have a false-positive rate, mainly due to radiation-induced inflammation causing FDG avidity. Among four NI-RADS2 primary disease cohort patients who did not show clinical disease progression at 1 year but turned out to be positive for the disease on HPR and were PET avid, it is suggested that, in combination with CECT, PET may help in raising the suspicion level of disease in this group of NI-RADS2, where NI-RADS category can be upgraded to NI-RADS3 and biopsy in such cases can be used as a problem-solving tool, hence prevailing the management recommendation in NI-RADS2 category of suggesting PET/CECT where only CECT was done. Here there is an opportunity to understand further that PET/CECT can be used for close interval follow-up in such stage III/IV patients to pick up residual disease/recurrence early.

Post-treatment CECT was better able to raise the suspicion of disease in NI-RADS3 and NI-RADS4 categories of both cohorts as compared to PET, hence subjecting these cases to early intervention for further evaluation to rule out residual disease/recurrence. Here imaging was better able to pick up disease progression in NI-RADS3 and NI-RADS4 cases as compared to clinical follow-up. This is an area which can be explored further to assess if the statistical significance of CECT performs better over clinical disease status on follow-up.

This is the first single institutional study with a large number of a uniform cohort of stage III and IV cases of the oropharynx and other cancer (hypopharynx and larynx) patients who underwent CRT and PET/CECT imaging as the first post-treatment scan (17) (with 435 cases in the primary disease cohort and 412 cases in the node Disease cohort included). We had a good number of cases with 1-year follow-up and low dropout cases, which helped evaluate NI-RADS’s performance in such a uniform cohort. Nearly all the first-post treatment PET/CECT scans were performed at around 8 weeks.

Since this study was for patients who underwent CRT as primary treatment, only advanced-stage patients were included in this study, and we had to exclude stages I–II cases. Patients with an incomplete response should undergo more frequent clinical and imaging surveillance than patients with a complete response. Furthermore, the true benefits of NI-RADS application with PET/CECT surveillance guidelines for the early detection of recurrent or residual disease and the potential impact on patients’ overall survival require further investigation, preferably with an additional long-term prospective study. Lastly, in our study, only stages III and IV cases of patients from a single institution were included in the study cohort. Post-operative cases who underwent CRT later were also excluded from the study. Therefore, the results may not be comparable or universalized if all stages and post-operative cases are included in the study cohort. The prognostic value of radiological extranodal extension was also studied in this cohort, where it was proved that rENE is an independent prognostic sign for survival in patients with LAHNSCC treated radically with CCRT that may be employed as a potential predictive marker for responsiveness to treatment, allowing for the stratification of patients into responders and non-responders (8, 29).

The NI-RADS was originally developed for surveillance contrast-enhanced CT imaging with or without PET in patients with treated HNC (30). The MRI-specific NI-RADS lexicon was soon published in view of superiority of MRI in evaluating the skull base, sinonasal region, nasopharynx, salivary glands, orbits, and especially for assessing perineural spread. The NIRADS has seen significant evolution over the last few years with the last revised version of PET/CT category descriptors, imaging lexicon, and management guidelines being published in January 2021, followed by the MRI lexicon in November 2021. We have employed the NI-RADS version of 2018 for this study. In areas where MRI is not routinely available, CT descriptors are the mainstay for reporting post-treatment surveillance imaging. Studies comparing the performance of PET vs. CT vs. MRI should be undertaken to determine the advantage of one imaging modality over the others in surveillance imaging. There will be greater developments in the protocols and reporting lexicon of head neck cancer surveillance imaging as more diverse original research works surface with time.

## Conclusion

The accuracy with which the NI-RADS lexicon performed in our study at node sites was better than that at the primary site. There is a great scope of research to understand if CECT performs better over clinical disease status in NI-RADS3 and NI-RADS4 categories. Further research should be carried out to understand if PET/CECT can be used for close-interval follow-up in stage III/IV NI-RADS2 cases.

## Data availability statement

The original contributions presented in the study are included in the article/**Supplementary Material**. Further inquiries can be directed to the corresponding author.

## Author contributions

Study concept: AM. Study design: AM, HU. Data acquisition: AM, HU. Quality control of data algorithms: AM, HU, SS, NS, RV. Statistical analysis: AM, HU Manuscript preparation: all authors. Manuscript editing: AM, HU, SS. Manuscript review: all authors. All authors contributed to the article and approved the submitted version.
