# Supplementary figures and images for: Response assessment of post-treatment head and neck cancers to determine further management using NI-RADS (Neck Imaging Reporting and Data System): a subgroup analysis of a randomized controlled trial
Source: Front Oncol. 2023 Sep 21;13:1200366. doi: 10.3389/fonc.2023.1200366 (PMC10552531; doi:10.3389/fonc.2023.1200366)

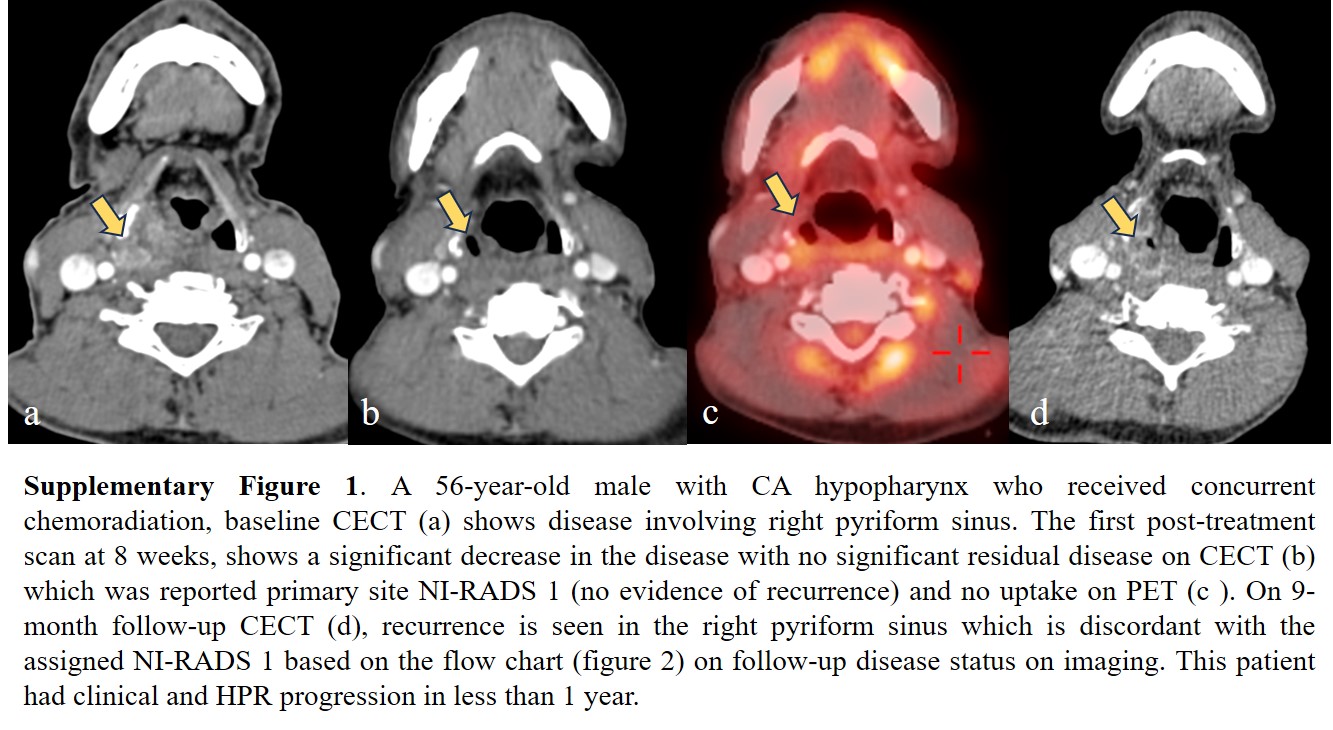

Supplement: Supplementary file 1 [file Image_1.jpg]

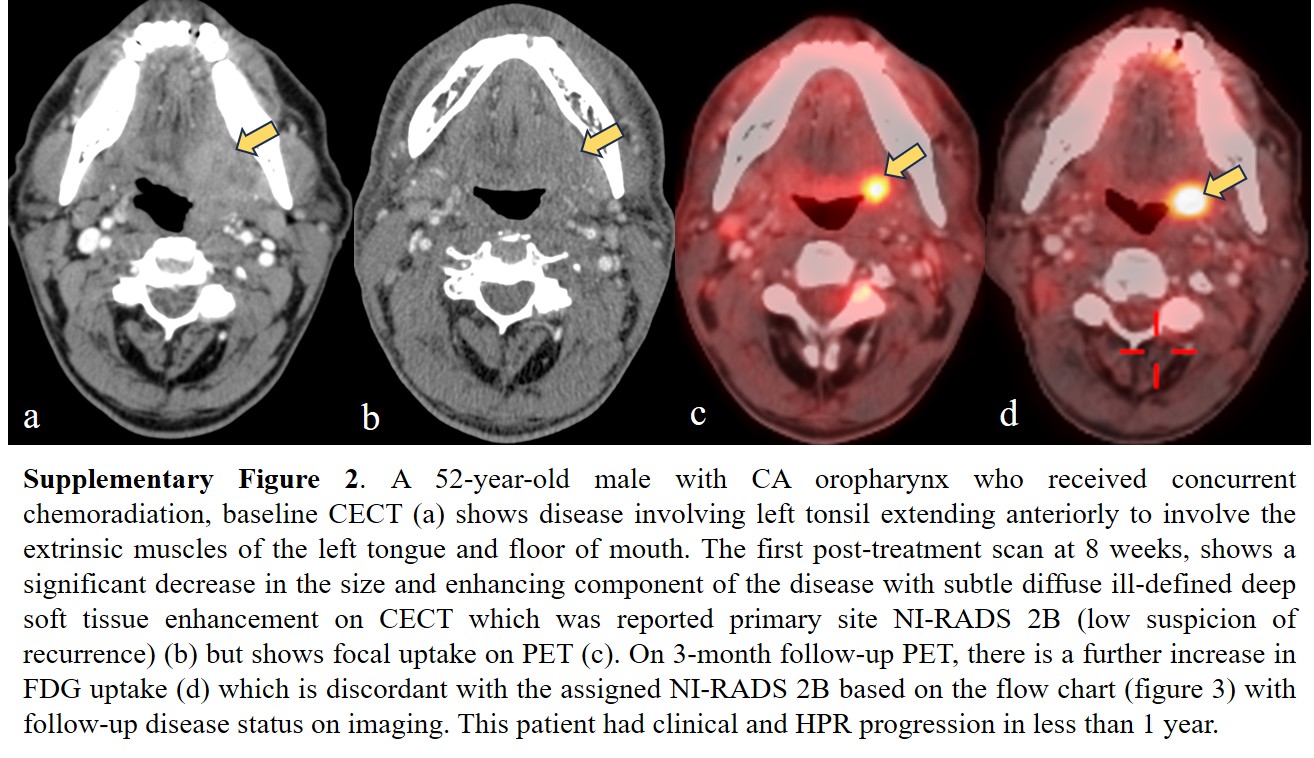

Supplement: Supplementary file 2 [file Image_2.jpg]

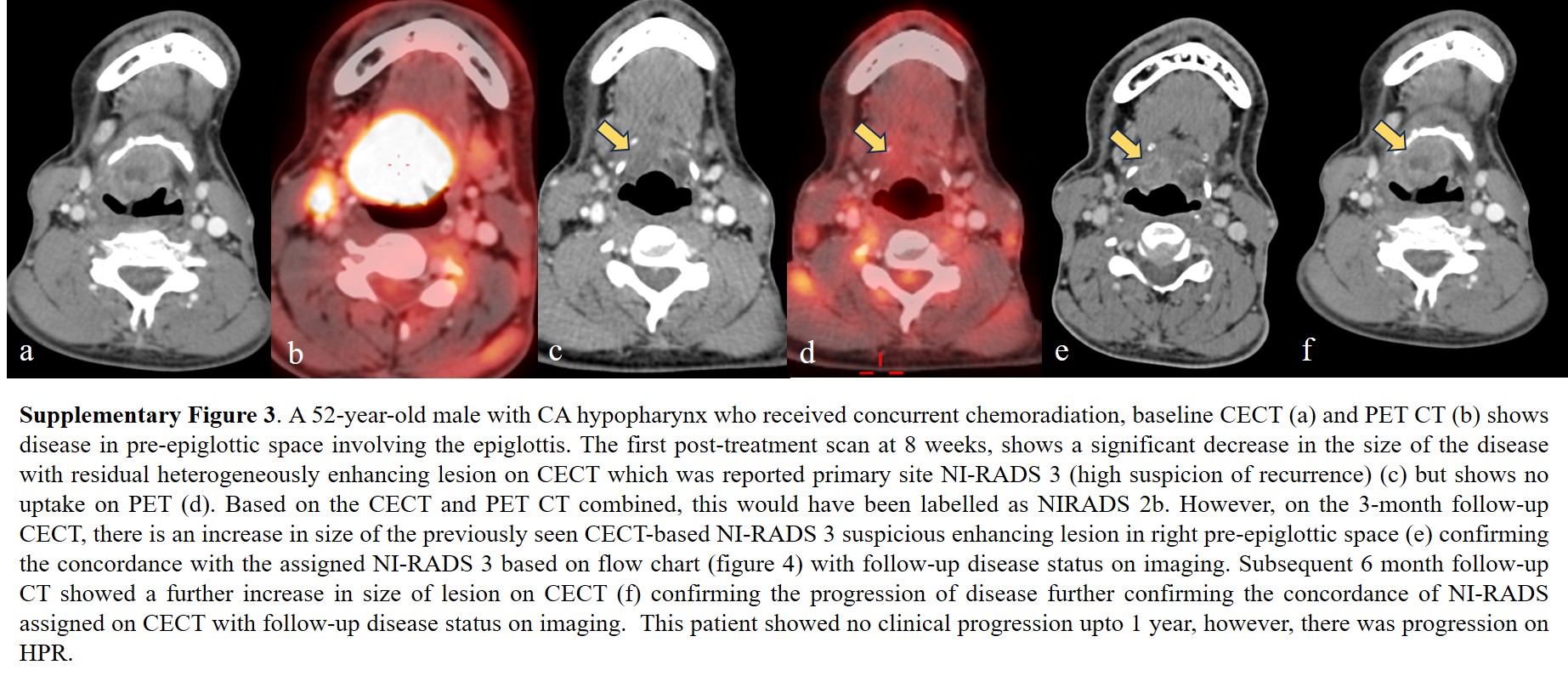

Supplement: Supplementary file 3 [file Image_3.jpg]

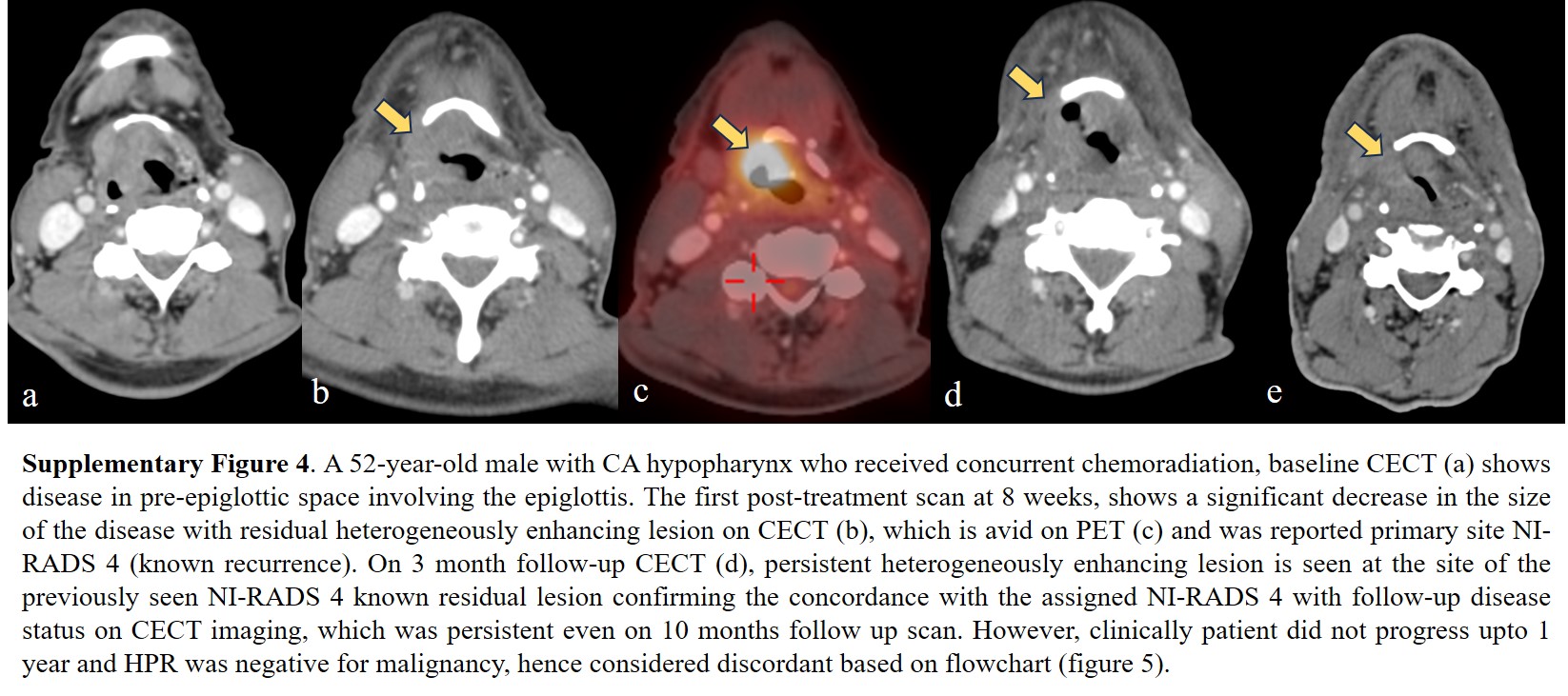

Supplement: Supplementary file 4 [file Image_4.jpg]

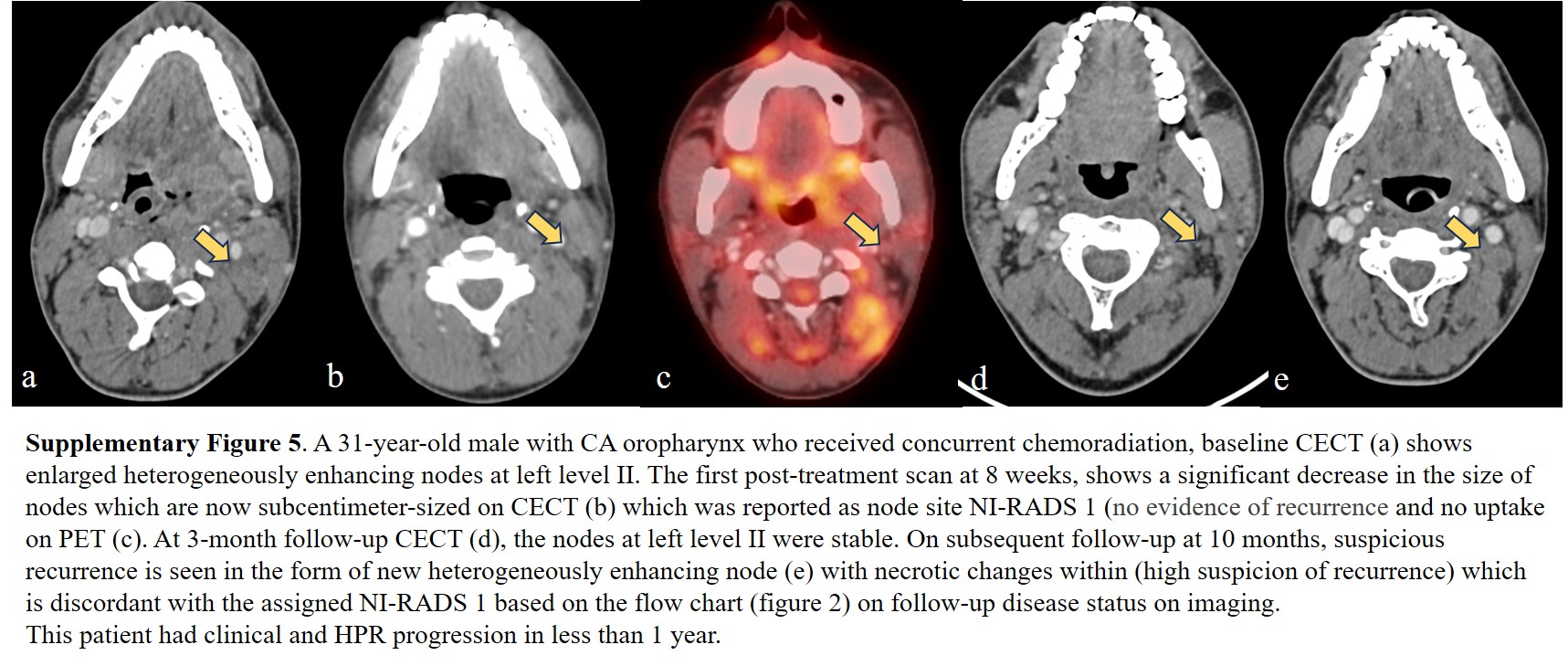

Supplement: Supplementary file 5 [file Image_5.jpg]

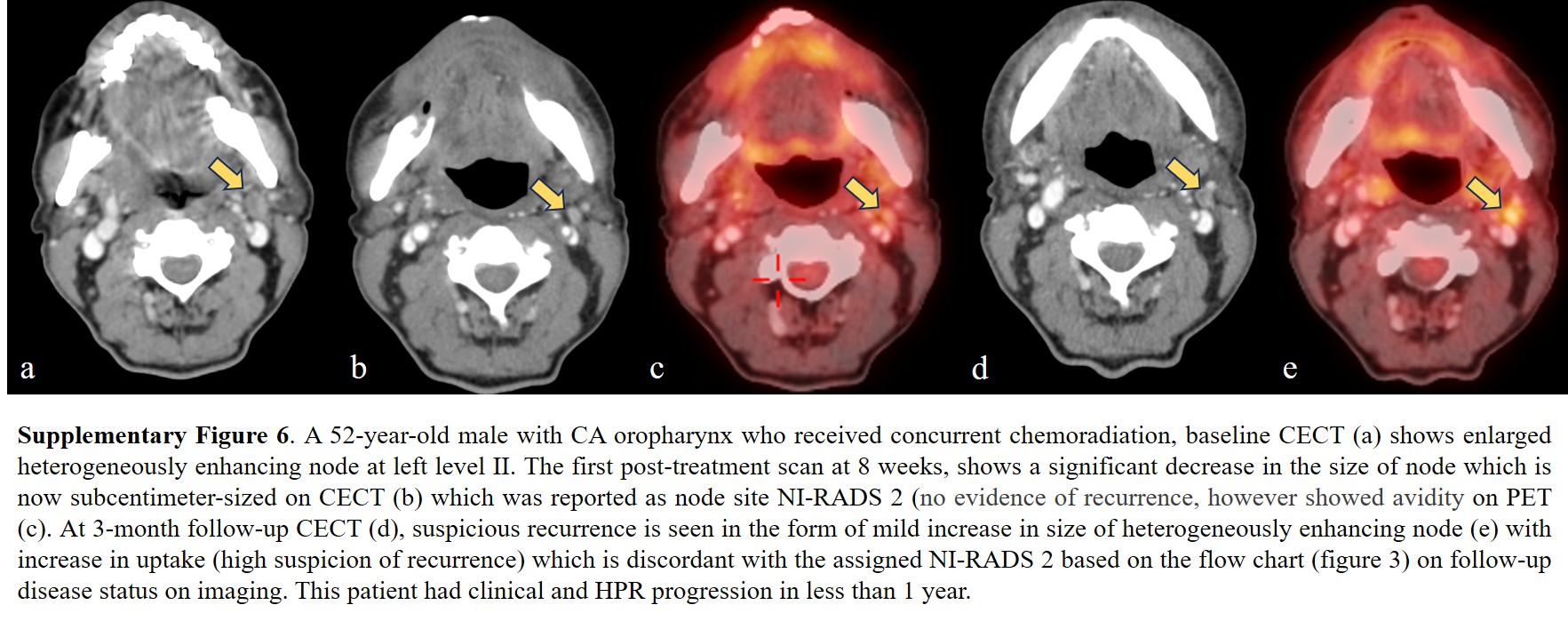

Supplement: Supplementary file 6 [file Image_6.jpg]

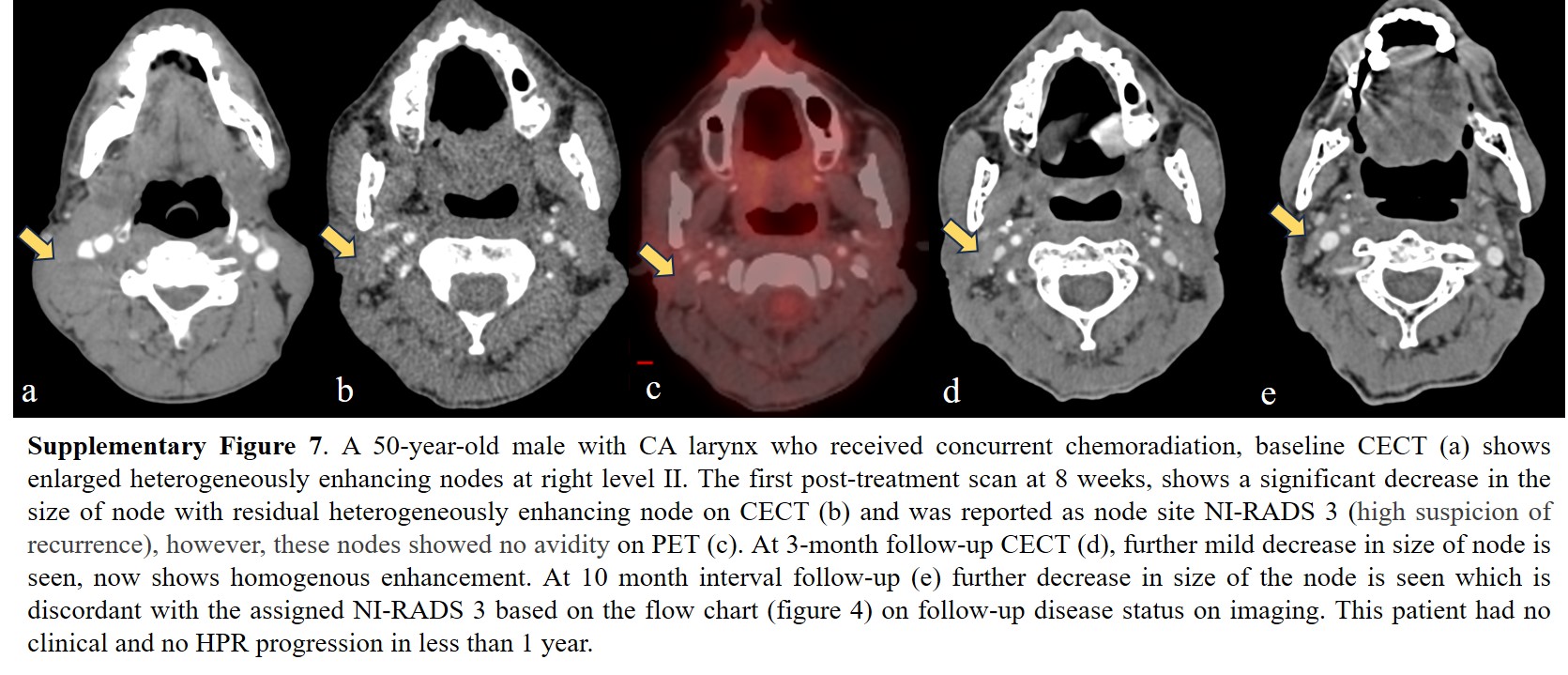

Supplement: Supplementary file 7 [file Image_7.jpg]

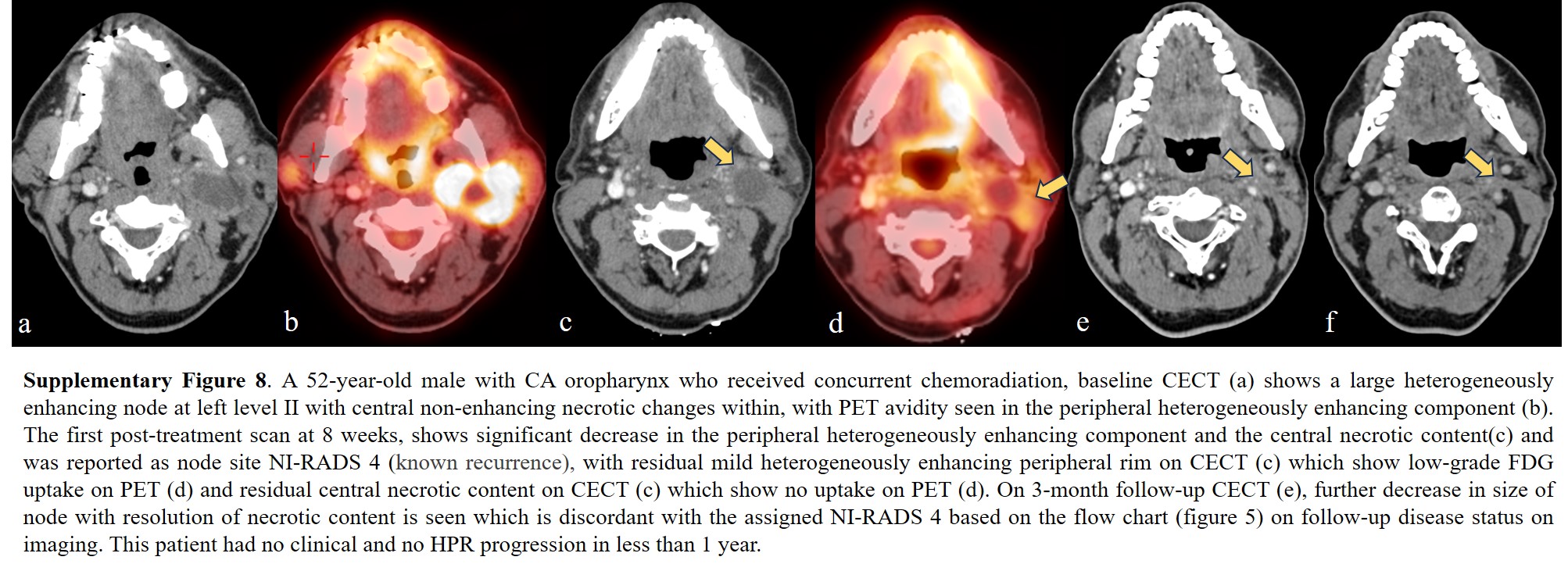

Supplement: Supplementary file 8 [file Image_8.jpg]
